# An Online Digital Imaging Excitation Sensor for Wind Turbine Gearbox Wear Condition Monitoring Based on Adaptive Deep Learning Method

**DOI:** 10.3390/s24082481

**Published:** 2024-04-12

**Authors:** Hui Tao, Yong Zhong, Guo Yang, Wei Feng

**Affiliations:** 1 Shien-Ming Wu School of Intelligent Engineering, South China University of Technology, Guangzhou 510640, China; 2 National and Local Joint Engineering Research Center for Industrial Tribology and Lubrication Technology, Guangzhou 510535, China; 3 Department of Industrial and Manufacturing Systems Engineering, The University of Hong Kong, Hong Kong 999077, China; 4 Guangzhou Mechanical Engineering Research Institute Co., Ltd., Guangzhou 510535, China

**Keywords:** digital imaging, excitation sensor, magnetic particles, lubricating oil, U-Net network, watered algorithm, MTF network

## Abstract

This paper designed and developed an online digital imaging excitation sensor for wind power gearbox wear condition monitoring based on an adaptive deep learning method. A digital imaging excitation sensing image information collection architecture for magnetic particles in lubricating oil was established to characterize the wear condition of mechanical equipment, achieving the real-time online collection of wear particles in lubricating oil. On this basis, a mechanical equipment wear condition diagnosis method based on online wear particle images is proposed, obtaining data from an engineering test platform based on a wind power gearbox. Firstly, a foreground segmentation preprocessing method based on the U-Net network can effectively eliminate the interference of bubbles and dark fields in online wear particle images, providing high-quality segmentation results for subsequent image processing, A total of 1960 wear particle images were collected in the experiment, the average intersection union ratio of the validation set is 0.9299, and the accuracy of the validation set is 0.9799. Secondly, based on the foreground segmentation preprocessing of wear particle images, by using the watered algorithm to obtain the number of particles in each size segment, we obtained the number of magnetic particle grades in three different ranges: 4–38 µm, 39–70 µm, and >70 µm. Thirdly, we proposed a method named multidimensional transformer (MTF) network. Mean Square Error (MSE), Root Mean Square Error (RMSE), and Mean Absolute Error (MAE) are used to obtain the error, and the maintenance strategy is formulated according to the predicted trend. The experimental results show that the predictive performance of our proposed model is better than that of LSTM and TCN. Finally, the online real-time monitoring system triggered three alarms, and at the same time, our offline sampling data analysis was conducted, the accuracy of online real-time monitoring alarms was verified, and the gearbox of the wind turbine was shut down for maintenance and repair.

## 1. Introduction

The wear particles in the oil, as products of relative motion on the surface of mechanical components, contain a lot of information on the state of the machine [1,2]. Usually, two methods of using oil monitoring technology are online monitoring and offline monitoring to observe wear particles, and the main technologies for offline monitoring include spectral and wear particle analysis [3,4]. Spectral analysis is based on the spectral lines of various atoms to determine which chemical elements are contained in the oil and the concentration of these elements and to determine the wear condition of various components. The papers [5,6] have developed instruments for collecting spectral data and analyzing the data using different methods, but the size of the collected wear particles is within 10 μm, so it is impossible to collect large wear particles in some fault states. Wear particle analysis technology [7] mainly enables wear particles to deposit on transparent substrates according to their size under the action of a magnetic field and then to observe and analyze the wear particles through optical or electron microscopy. In 1976, the paper [8] developed a commercial analytical wear particle. Subsequently, in 1977, wear particle technology was introduced in China, greatly enhancing the understanding of wear mechanisms. G. Chen et al. [9] used demography analysis to obtain the percentage of various wear particles in engine oil, but overall, offline oil monitoring [10] is conducted after collecting samples during the experimental process, which involves a large number of human factors, resulting in poor real-time performance.

After collecting the wear particle images, X. Zhu et al. [11] first performed wavelet transform on the images and then used two-dimensional wavelet decomposition and reconstruction to construct four sets of low-frequency and high-frequency images for processing. Finally, the low-frequency images were subjected to wavelet transform reciprocating construction until denoising was completed. This method considers the detailed information of the image, but wavelet denoising is mainly suitable for processing Gaussian noise, and its adaptability is not comprehensive enough. W. Cao et al. [12] used 2D-VMD to decompose the noisy image into some sub-modes including the original information of the wear image, while other sub-modes include background noise. After removing the sub-modes with background noise, the wear image was reconstructed. The decomposed model can better preserve the edge and other detailed information of the wear image and play a good role in removing the edge noise on the left and right sides of the image. W. Cao et al. [13] proposed a WVBOD image denoising model, which integrates three denoising methods and fully utilizes the advantages of the three to remove Gaussian noise and some surrounding bubbles in the wear image, thus preserving the main information of the abrasive. W. Zhou et al. [14] used Principal Component Analysis (PCA) to select wear particle feature parameters and improved the LS-SVM classifier based on a genetic algorithm, resulting in an increase in wear particle classification accuracy from 82.5% to 95%. L. Qiu et al. [15] proposed a wear particle image recognition method based on a support vector machine, which applied the superiority of SVM in small sample classification to wear particle image recognition and achieved good results. W. Yuan, K. Chin, M. Hua et al. [16,17] proposed an adaptive SVM recognition model based on an improved PSO algorithm and established the optimal adaptive SVM model by optimizing penalty parameters and kernel functions.

To preserve the information on online wear images, T. Wu et al. [18] eliminated the interference of light and dark fields in the wear images by using morphological black hat operations and H-minima transformation. Then, the Otsu threshold method was used to obtain the threshold, and finally, the watershed algorithm was used to separate and effectively obtain the wear particles in the wear images that were too bright and too dark due to reflected light irradiation. W. Wu et al. [19] used an adaptive Canny operator to perform preliminary segmentation on the bilaterally filtered, enhanced wear particle image, then used a histogram similarity measure to distinguish between wear particles and background, and finally filled the contour of the wear particles to obtain more accurate wear particle segmentation. At present, the academic community mainly studies how to process and optimize online wear particle images, but there is little research on the overall processing process of online wear particle images, that is, how to convert irregular data such as online wear particle images into regular data and then into wear indicators, to diagnose the wear condition of the equipment. Lakshmi, H.R. et al. [20] present an effective adaptive reversible image watermarking. The approach utilizes the selection of an optimal location for embedding according to entropy, where the appropriate threshold for entropy selection is taken care of by the particle swarm optimization (PSO) algorithm. A technique in integer wavelet transform (IWT) domain is proposed, further discrete cosine transform (DCT) and singular value decomposition (SVD) are hybridized for embedding process in all the chosen blocks, and the fractal-encrypted watermark bits are integrated into the coefficients of the image using the average proximity coefficient.

The traditional foreground segmentation algorithm for wear particle images has the following problems: due to the influence of high temperature on the lubricating oil during the operation or detection of mechanical equipment, it will dissolve or mix with air during the circulating flow stage, resulting in a large number of bubbles [21,22]. This makes it impossible to avoid the interference of bubbles in the capture process of wear particle images, and traditional image segmentation algorithms misjudge the bubbles in the wear particle images as wear particles for analysis. The threshold-based image segmentation algorithm cannot distinguish the threshold of bubbles and abrasives, and all bubbles and abrasives are segmented and recognized as abrasives, resulting in abnormal analysis. With the improvement in computing power, an increasing number of artificial intelligence algorithms have been widely applied, according to Suvizi. A et al. [23] proposed a parallel computational architecture based on the concept of cellular automata to accelerate the numerical solution of the steady-state water distribution network model, and the performance of the proposed method was compared with EPANET software (EPANET V2.0) for networks with different complexities and topologies. Zarreh. M et al. [24] present a mathematical model using game theory for the pricing of drinking water in a competitive environment comprising a Public Water System (PWS) and a Bottled Water Plant (BWP) under government intervention. This paper adopts a dynamic approach to address the time-dependent nature of precipitation and water demand, incorporating uncertainty in the tap water supply. The study also introduces models for peak and volumetric water pricing, deriving several key corollaries through parametric analysis, and presents a case study, modeled on real-world scenarios, to validate the proposed model.

This paper proposes a method for diagnosing the wear condition of mechanical equipment based on online wear particle images. Firstly, a foreground segmentation preprocessing method based on the U-Net network can effectively eliminate the interference of bubbles and dark fields in online wear particle images, providing high-quality segmentation results for subsequent image processing, A total of 1960 wear particle images were collected in the experiment, the average intersection union ratio of the validation set is 0.9299, and the accuracy of the validation set is 0.9799. Secondly, based on the foreground segmentation preprocessing of wear particle images, by using the watered algorithm to obtain the number of particles in each size segment, we obtained the number of magnetic particle grades in three different ranges: 4–38 µm, 39–70 µm, and >70 µm. Thirdly, we proposed a method named multidimensional transformer (MTF) network, MSE, RMSE, and MAE evaluation indexes are used to obtain the error, and the maintenance strategy is formulated according to the predicted trend. The experimental results show that the predictive performance of our proposed model is better than that of LSTM and TCN. Finally, the online real-time monitoring system triggered three alarms, and at the same time, our offline sampling data analysis was conducted three times, the accuracy of online real-time monitoring alarms was verified, and the equipment was disassembled for maintenance and repair.

## 2. Materials and Methods

### 2.1. Working Principle and Logical Control Process of the Digital Imaging Excitation Sensor (DIES)

When the oil flows into the sensor and passes through the high-intensity magnetic field generated by the two magnetic poles, the ferromagnetic wear particles in the oil will be adsorbed on the glass surface by the magnetic field. The image of the wear particles will be collected through optical lens imaging and a CMOS industrial camera and processed and analyzed using specific artificial intelligence image recognition algorithms to achieve the real-time monitoring of the size, morphology, and other parameters of the ferromagnetic particles in the oil. It can further monitor the wear development process of easily worn parts of mechanical equipment (such as gears and bearings). Once the wear reaches a certain threshold, an alarm function can be achieved which can effectively avoid unplanned downtime and secondary damage to the equipment.

**Figure 1:**
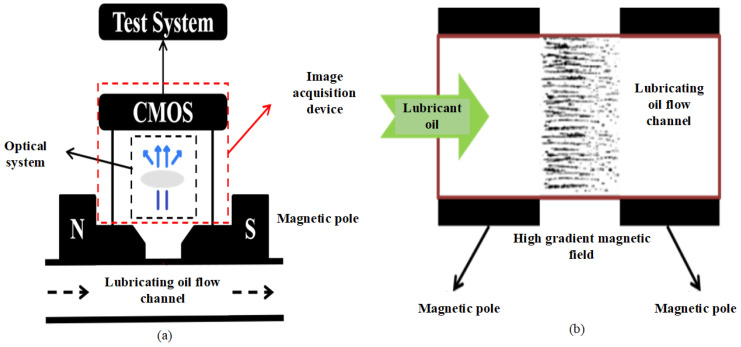
(**a**) Schematic diagram of the imaging structure principle of wear particles in oil. (**b**) Imaging of wear particles in oil.

The working logic flowchart of the sensor is based on Figure 1 when the sensor starts, the LED lights up, and the micro pump starts flushing mode, and then, the micro pump switches to the adsorption pump speed, and the controller loads magnetic force through the excitation coil to adsorb wear particles, and next, industrial cameras collect images of wear particles and perform image processing, analysis, and storage. Secondly, the micropump switches back to flushing mode to remove magnetic force and release wear particles. Finally, the micropump stops, the LED light turns off, and, at the same time, the sensor waits for the next acquisition.

### 2.2. U-Net Network Preprocessing Method

**Figure 2:**
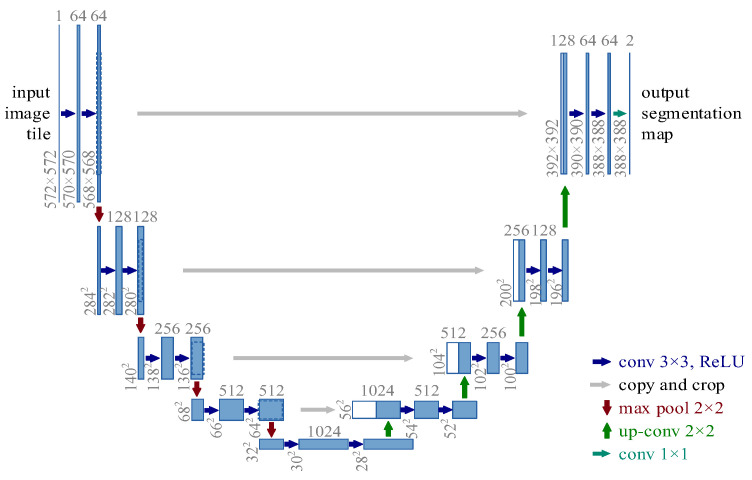
U-Net network structure.

The U-Net network structure diagram is shown in Figure 2. The U-Net network architecture [25] is divided into two main parts: compression path and expansion path. The former uses continuous convolution and pooling layers to reduce the dimensionality of the feature space, usually including two unfilled 3 × 3 convolutions followed by ReLU activation and batch normalization, followed by 2 × 2 max pooling to reduce the dimensionality of the feature map and increase the number of feature channels in each step. The latter uses 2 × 2 sampling convolution to double the size of the feature map, connects the result with the corresponding cropped compressed path feature map, and then performs two 3 × 3 convolutions, followed by ReLU activation. The output of the network is reduced to the required number of classifications by 1 × 1 convolution of the final feature map. The design of U-Net highlights the advantages of fully convolutional networks with their unique U-shaped structure, especially for fine foreground segmentation tasks. It can effectively combine the contextual information of different resolutions to ensure the accuracy of segmentation results at edge details and solve the bubble interference problem in traditional image segmentation methods.

### 2.3. Marked Watershed Algorithm

**Figure 3:**
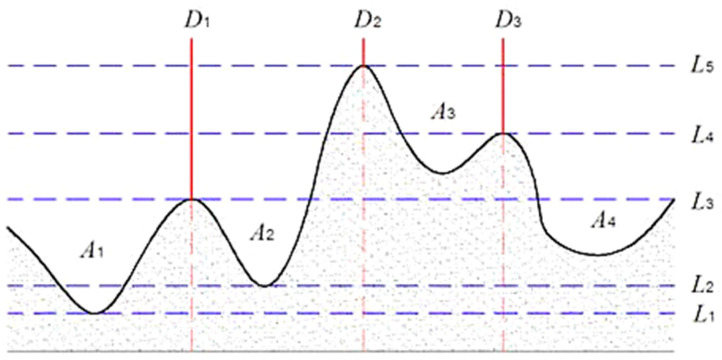
Schematic diagram of watershed algorithm.

Figure 3 is a schematic diagram of the two-dimensional watershed algorithm [26], where the black solid line represents the terrain, which includes four basins A1~A4; the blue dashed lines L1~L5 represent different water surface heights. When the waterline rises to L1, water begins to enter Basin A1. As the waterline rises to L2, Basin A2 also begins to flood. When the water level reaches L3, the water in Basin A1 and Basin A2 is about to meet, and Dam D1 is constructed at this time. When the water level rises to L4, the water in A3 and A4 is about to meet, and Dam D3 is being built at this time. Finally, when the waterline reaches L5, the water from Basin A2 and Basin A3 meet, and Dam D2 is constructed. This separates the four basins of the model through dams D1, D2, and D3.

The extraction of image markers includes foreground (abrasive) marker images and background marker images. Firstly, the threshold t is set, then all local minimum points are detected in the gradient image, and whether each minimum point is greater than the threshold t is determined. All points that are greater than the threshold t are marked, as Equation (1):

(1) $$g^{imark}\;\left(x,y\right)=1\;(if\;{H_{b}}^{\;\left(rec\right)\;}\;\left(x,y\right)>t)\;else\;if\;g^{imark}\;\left(x,y\right)=0\;\left(if\;{H_{b}}^{\;\left(rec\right)\;}\;\left(x,y\right)\;\leq t\right)\;$$

In the above Equation (4), ${H_{b}}^{\;\left(rec\right)\;}\;\left(x,y\right)\;$ represents the reconstructed image after composite opening and closing, and $g^{imark}\;\left(x,y\right)\;$ represents the foreground marker image.

The morphological gradient image $g\left(x,y\right)$ obtained after background subtraction is used as the original image for watershed transformation, and the minimum value forcing technique in mathematical morphology is used to modify the original segmented image. The extracted foreground $g^{imark}\;\left(x,y\right)\;$ and background markers $g^{emark}\;\left(x,y\right)$ are used to modify the local minima of the original gradient image. Therefore, in the modified gradient image, only the corresponding binary-labeled image that is not zero is forced to have local minima. The modified gradient image $g^{ws}\;\left(x,y\right)\;$ is represented by Equation (2):

(2) $$g^{ws}\;\left(x,y\right)=Mmin\;\left[g\;\left(x,y\right),g^{imark}\;\left(x,y\right)\;/g^{emark}\;\left(x,y\right)\;\right]$$

Among them, Mmin() represents the minimum imposed operation. Finally, the watershed algorithm is used to the achieve automatic segmentation of wear particle images as in Equation (3):

(3) $$\mathrm{SEG}\;\left(f\right)=\mathrm{WS}\;\left(g^{ws}\;\left(x,y\right)\right)\;$$

The formula $\mathrm{WS}\;\left(g^{ws}\;\left(x,y\right)\right)\;$ represents watershed operation, and label control is the key to the watershed segmentation algorithm, which directly affects the final segmentation effect. For the image of wear particles, they are in an oil environment with rough and irregular surfaces, and it is difficult to determine the boundaries between particles from the gradient map. The gradient map is not as suitable as the original image for watershed segmentation. At the same time, it is noted that, under the action of a magnetic field, the wear particles are distributed along the direction of the magnetic field line, presenting a “chain” shape. The majority of the wear particles are connected up and down, with a few being connected left and right.

### 2.4. Multidimensional Transformer Network (MTF)

#### 2.4.1. Multidimensional Data Preprocessing and Multi-Head Attention Module

This module is employed for data preprocessing to extract redundant and complementary features related to oil wear. By utilizing Equation (4), the initial data are averaged on a minute basis to acquire daily data.

(4) $${\bar{x}}_{i}=\sum_{i=1}^{T}x_{i}\;/\;T$$

*x_i_* represents the monitoring value at the i-th instance, while ${\bar{x}}_{i}$ corresponds to the average value over *T* instances. To process the raw data, a numerical accumulation method is employed, as demonstrated in Equation (5).

*X_i_′* = *diff* (*log* (*x_i_*)) (5)

**Figure 4:**
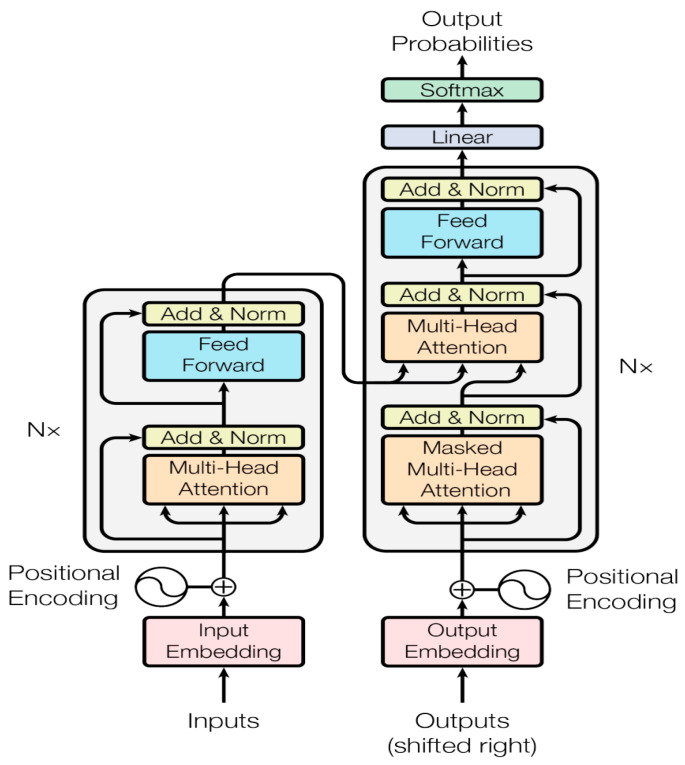
The principle of the multi-head attention mechanism.

The *diff* () function is a mathematical tool for calculating the derivative of a given function. Furthermore, the multi-head attention mechanism is employed to improve the self-attention learning capability. This involves mapping *Query*, *Key*, and *Value* to distinct subspaces within a higher-dimensional space while maintaining a consistent total parameter count. The module then integrates attention information from these various subspaces, as depicted in Figure 4. The main goal of the multi-head attention mechanism is to improve the expressive power of the model and the ability to capture the different levels of information.

Additionally, the process of calculating attention mapping inputs across various representation spaces can be described as follows:

*MultiHead* (*Q*,*K*,*V*) = *Concat* (*head*_1_,…, *heads) W^O^* (6)

*Head* = *Attention (QW_i_^Q^*, *KW_i_^Q^*, *VW_i_^Q^)* (7)

where $W_{i}^{Q}\in R^{d_{model}\times d_{k}}$,$\;W_{i}^{K}\in R^{d_{model}\times d_{k}}$, $W_{i}^{V}\in R^{d_{model}\times d_{v}}$, and $W^{O}\in R^{hd_{v}\times d_{model}}$. h represents the number of attention heads, while $\;d_{k}$, $d_{v}$, and $d_{model}$ are predefined coefficients. The attention mechanism is calculated using the weight matrix (Q, K and V) and the coefficient matrix (*W^Q^*, *W^K^* and *W^V^*). x refers to thinking machines, with each attention head containing multiple pairs of $Q_{0}$ and $W_{0}^{Q}$. By conducting n self-attention calculations using distinct weight matrices, n different Z-matrices can be obtained. Furthermore, these n matrices are combined into a single matrix.

#### 2.4.2. Positional Encoding and Encoder–Decoder Module

The primary components of this module are position encoding and the encoder–decoder. Position encoding in the transformer model serves to represent sequence order, aiding the model in determining the position of each element in the sequence and the relative distance between features. The vector computation for position encoding is demonstrated in Equations (8) and (9):

(8) $$PE_{\;\left(pos,2i\right)\;}=\sin\;\left(pos/10000^{2i/d_{model}}\right)\;$$

(9) $$PE_{\;\left(pos,2i+1\right)\;}=\cos\;\left(pos/10000^{2i/d_{model}}\right)\;$$

*pos* denotes the position. $PE\left(pos+k\right)$ can be expressed as a linear function of $PE\left(pos\right)$:

cos (*α* + *ß*) = cos (*α*) cos (*ß*) − sin (*α*) sin (*ß*) (10)

sin (*α* + *ß*) = sin (*α*) cos (*ß*) + cos (*α*) sin (*ß*) (11)

#### 2.4.3. Prediction and Maintenance Strategy Module

We employed Mean Square Error (MSE), Root Mean Square Error (RMSE), and Mean Absolute Error (MAE) as assessment metrics for the model’s predictive capabilities, with their respective calculations provided in Equations (12)–(14). Lower error values indicate superior prediction performance.

(12) $$Error_{MSE}=\frac{1}{m}\sum_{i=1}^{m}\;\left(y_{i}-{\hat{y}}_{i}\right){\;}^{2}$$

(13) $$Error_{RMSE}=\sqrt{\frac{1}{m}\sum_{i=1}^{m}\;\left(y_{i}-{\hat{y}}_{i}\right){\;}^{2}}$$

(14) $$Error_{MAE}=\frac{1}{m}\left|y_{i}-{\hat{y}}_{i}\right|$$

## 3. Establishment of the Experimental Platform

The on-site testing site of this article is installed at a wind power plant in Guangdong, and the status monitoring of two wind turbines in the wind farm is carried out.

### 3.1. Information and Monitoring Indicators of Wind Power Gearbox Equipment

The device information table is shown in Table 1.

### 3.2. Engineering Testing Platform

**Figure 5:**
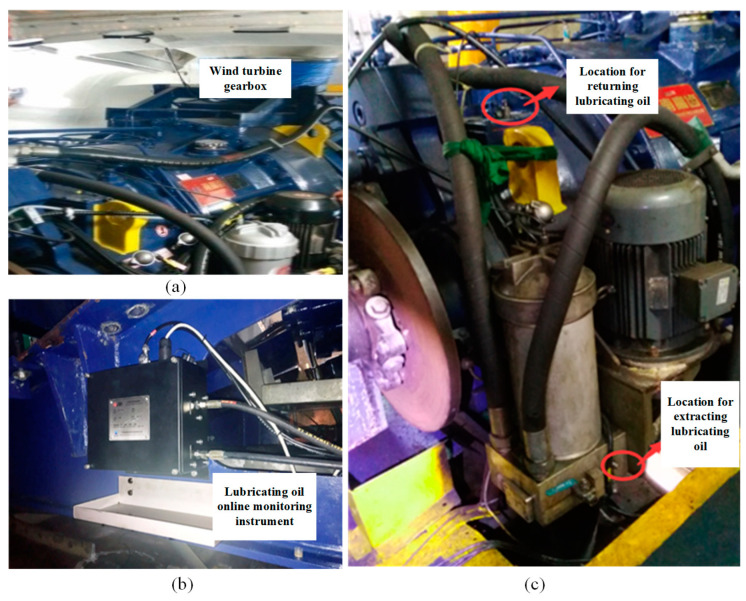
(**a**) Gearbox of doubly fed wind turbine generator; (**b**) the specific installation location of the sensing system; and (**c**) oil pick-up and return positions.

The gearbox of the doubly fed wind turbine is shown in Figure 5a, and the specific installation position of the sensing system is shown in Figure 5b.

(1) Oil sampling point: Before filtration, a sample is taken, and the G1/4 quick pressure measuring connector at the base of the circulating oil pump input filter element is replaced with a G1/4 three-way valve. It is divided into two oil circuits, one of which is installed back to the original pressure measuring connector, and the other is equipped with a G1/4 ball valve and connected to the high-pressure hose oil circuit to connect to the oil inlet of the oil online monitoring instrument. As shown in Figure 5c, a return point is present on the upper oil cover of the main engine, the original G1/4 quick pressure measuring joint is replaced with a G1/4 three-way joint, and it is divided into two oil circuits. One circuit is installed back to the original pressure measuring joint, and the other circuit is equipped with a G1/4 ball valve and connected to the high-pressure hose oil circuit to connect to the return port of the oil online monitoring instrument, as shown in Figure 5c.

## 4. Experiments and Results

### 4.1. U-Net Network and Watershed Algorithm

#### 4.1.1. Dataset Preparation

**Figure 6:**
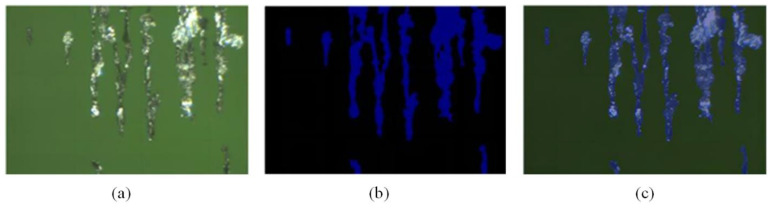
Presentation of the annotation effect of wear particle image data: (**a**) original image, (**b**) mask annotation, and (**c**) overlay effect.

Firstly, we use DIES to collect the images of various types of wear particles, such as those with bubbles, uneven distribution of light and dark, a large amount of oil sludge, individual wear particles, a large number of wear particles, different types of wear particles present simultaneously, and only normal wear particles. A total of 1960 wear particle images were collected in the experiment. Image annotation based on semantic segmentation uses LabelMe to annotate the foreground of wear images. Figure 6 shows the overall effect of wear image data annotation. The labeled wear particle images are divided into a training dataset of 1373, a testing dataset of 391, and a validation dataset of 195, based on a ratio of 70% for the training dataset, 20% for the testing dataset, and 10% for the validation dataset.

#### 4.1.2. Model Training

For the 1960 collected wear images, the input size of the images is set to 640 px in width and 450 px in length, with an epoch of 200, a learning rate of 0.02, and a batch size of 4. The method for reducing the learning rate is polynomial, and the optimization method is Stochastic Gradient Descent (SGD). The model parameters can be gradually adjusted during the training process to find local optimal solutions. This enables the model to better fit the training data.

To achieve better training results, data augmentation was carried out by randomly flipping, blurring, brightness, contrast, saturation, and color tone operations on wear images. The probability of brightness, contrast, saturation, color, and minimum truncation area ratio was set to 0.5. The color range was set to 18, and the aspect ratio was set to 0.33. At the end of the training, the loss function value of the training set is 0.0506.

#### 4.1.3. U-Net Preprocessing Results

The training results of the U-Net network model are as follows: the average intersection union ratio (MIOU) of the validation set is 0.9299, and the accuracy of the validation set (OACC) is 0.9799. The final evaluation indicators for image segmentation include an average accuracy rate (MACC) of 0.9805 and an average intersection-to-union ratio (MIOU) of 0.9329. The overall average indicators after segmentation are shown in Table 2. Among them, category 0 is the background after segmentation, and category 1 is the wear particles in the foreground after segmentation.

**Figure 7:**
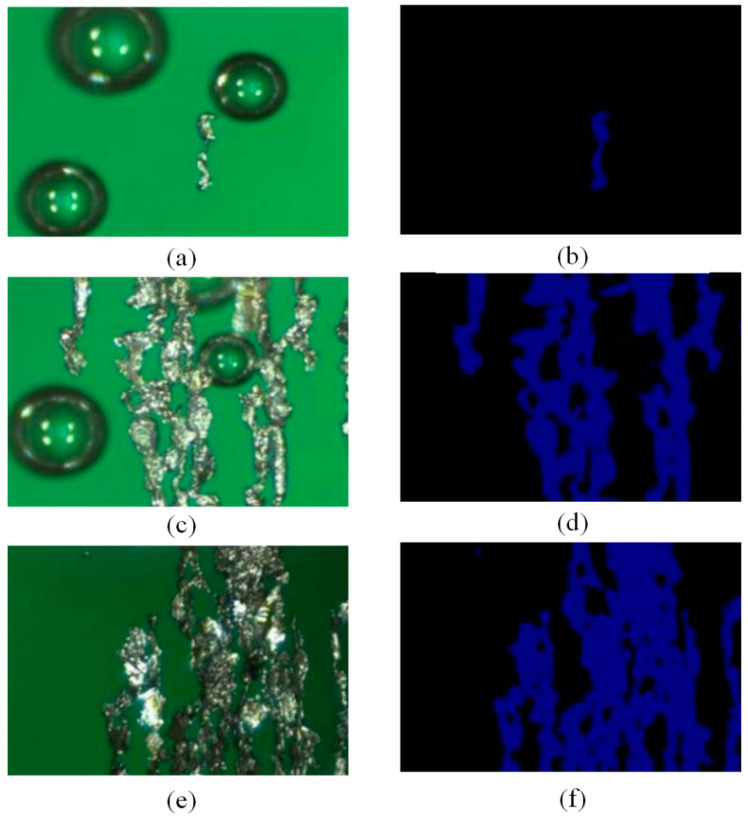
Segmentation effect images based on U-Net network training: (**a**) original image; (**b**) processed effect image; (**c**) original image; (**d**) processed effect image; (**e**) original image; and (**f**) processed effect image.

Figure 7 shows the segmentation results of effect images based on U-Net network training. Figure 7a,c,e show the image when U-net does not exist, and Figure 7b,d,f show the segmentation results of effect images based on U-Net network training.

#### 4.1.4. Watershed Feature Processing Results

**Figure 8:**
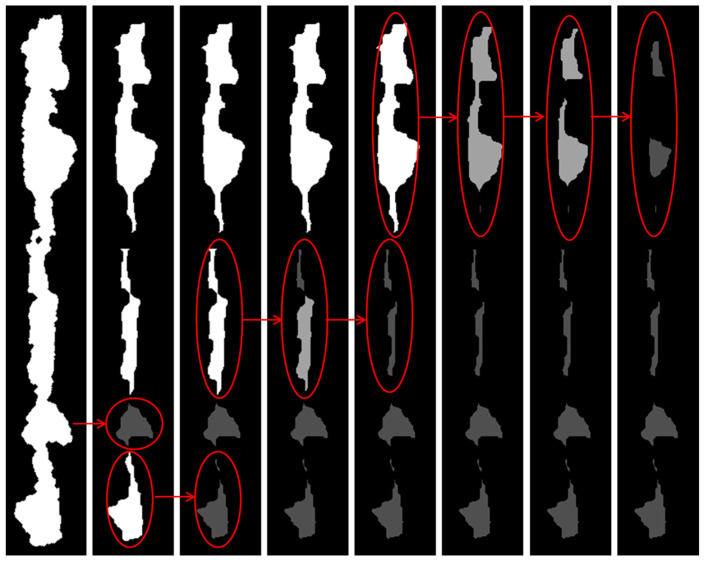
Foreground-marking process processing diagram.

Figure 8 was a schematic diagram of the foreground labeling process. After one corrosion, the abrasive chain was divided into four parts and sorted from top to bottom. The length-to-length ratio of the third part of the particles met the standard of seed points and was directly saved as seed points without further processing. The remaining three parts were processed separately. The fourth part was divided into two parts after one corrosion, both of which met the seed point standard and will not be processed after preservation. In the second part, after multiple corrosion operations, two seed points were finally obtained. After multiple corrosion operations, the first part ultimately obtained three seed points. The end was the final foreground marker point.

**Figure 9:**
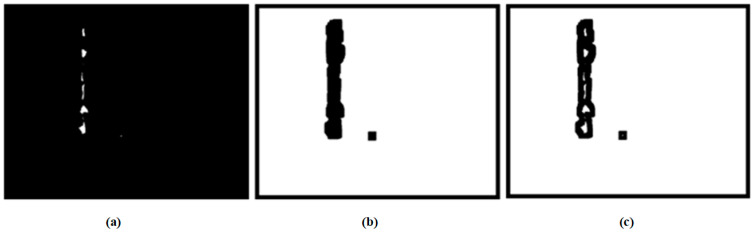
Mark combination diagram: (**a**) foreground markers; (**b**) background markers; and (**c**) mark combination.

The background area was obtained by inverting the foreground area, processing the border, and then enabling morphological corrosion. Foreground markers are overlaid with background markers to obtain the final marker image, as shown in Figure 9.

**Figure 10:**
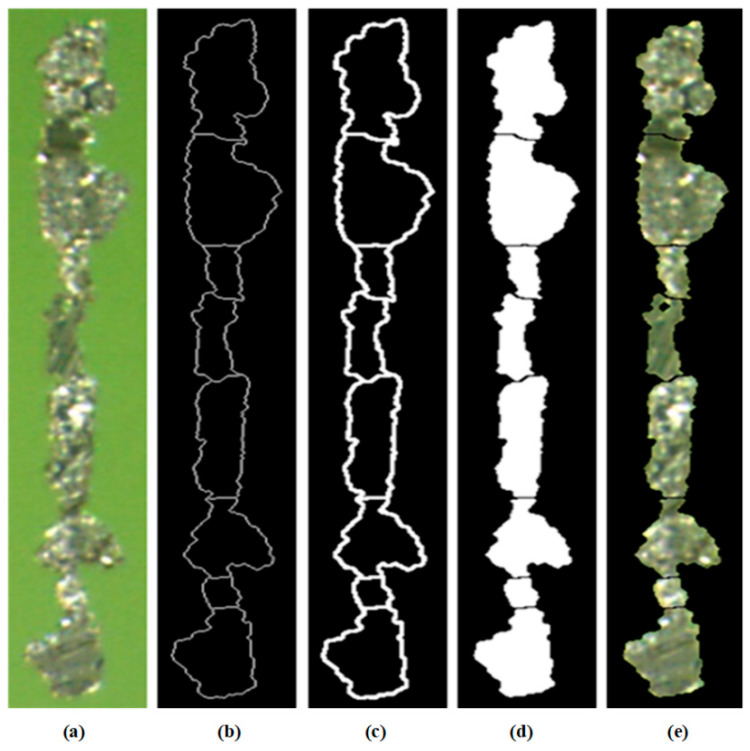
Effect image of watershed segmentation: (**a**) original image; (**b**) original segmentation line; (**c**) expanded segmentation line; (**d**) template image of wear particles; and (**e**) color image of wear particles.

The segmentation effect is shown in Figure 10. Figure 10a was the original image, Figure 10b was the original watershed segmentation line (with a line width of one pixel), Figure 10c was the result of morphological expansion in Figure 10b, Figure 10d was the final segmented template image of wear particles, and Figure 10e was the color image of segmented wear particles.

#### 4.1.5. Classification of Wear Particles in Lubricating Oil

Oil wear particles were analyzed based on the following two parameters:

1. Coverage area ratio

It refers to the ratio of the area occupied by wear particles in the wear particle image to the overall area of the image. This parameter can intuitively reflect the degree of contamination of the oil. The calculation formula is as follows, where R is the coverage area ratio, AF is the total number of pixels in the abrasive coverage area, and M and N are the length and width of the image, as in Equation (15).

(15) $$R=\frac{A_{F}}{M*N}$$

2. Grading and counting of wear particles

It refers to classifying wear particles according to the size of wear particles and calculating the number of wear particles in each category. At present, the particle size of wear particles is divided into six levels based on the equivalent circular diameter of the particles, including 4–6, 6–14, 14–21, 21–38, 38–70, and above, in micrometers (μm). The conversion formula for equivalent circle diameter and area, where D is the equivalent circle diameter and A is the abrasive area, is provided in Equation (16).

(16) $$D=\sqrt{\frac{4*A}{\pi }}$$

3. Extraction of Morphological Features of Single Large Wear Particle

The extraction of two-dimensional morphological feature parameters of wear particles was crucial for wear particle analysis, as it can obtain rich wear particle feature information, which can meet the classification needs of common types of wear particles. The two-dimensional morphological feature parameters of wear particles referred to the morphological feature parameters directly extracted from two-dimensional static wear particle images. The main feature parameters extracted in this article include the following:

a. Area of wear particles

The total number of pixels in the connected domain with a median of one in a binary graph.

b. Perimeter of wear particles

The total number of boundary pixels in a connected domain with a median of one in a binary graph.

c. Equivalent area circle diameter of wear particles

The abrasive area is converted to the equivalent circular area diameter according to Equation (8).

**Figure 11:**
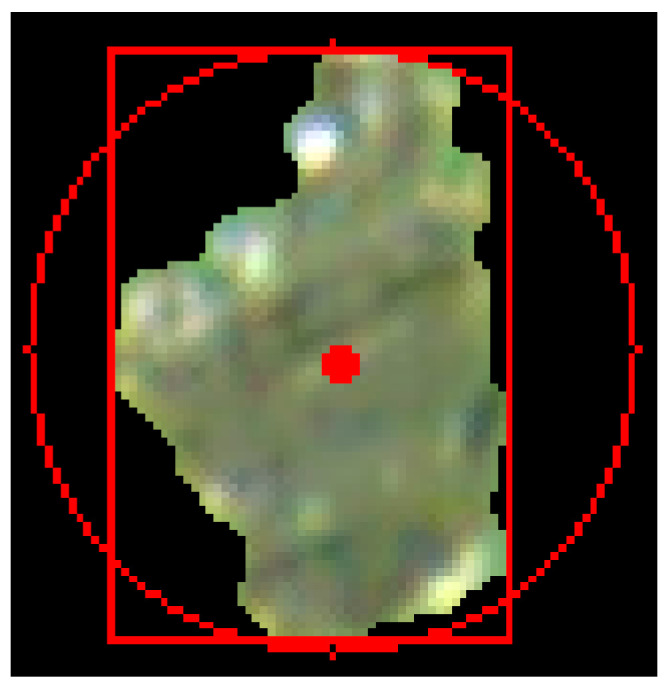
Individual large wear particles.

The long axis and short axis of the wear particles are the length and width of the smallest rectangle surrounding the target. This parameter can distinguish slender targets from approximately circular or square targets. Figure 11 shows a separately extracted particle, with the smallest rectangle surrounding it marked with a red rectangle and the center of mass of the wear particles marked with a red solid circle.

#### 4.1.6. Test Results of Oil Wear Particle Classification Experiment

**Figure 12:**
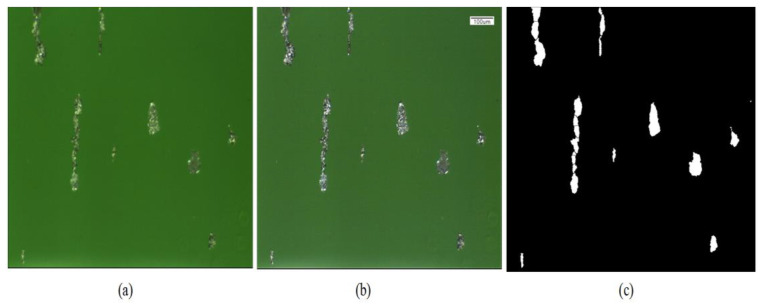
Wear particles pictures segmentation and grading of wear particles: (**a**) picture of wear particles before grading treatment. (**b**) picture of wear particles Preprocessed image and (**c**) Picture of wear particles after grading treatment.

The repeatability error of particles was an indicator of traditional shading particle counters. To test the stability of the software’s calculation data, the same wear particle image was repeatedly processed to analyze whether there were fluctuations in the resulting data. Specific operation included the following: randomly selecting an original wear particle image, with segmentation effect image being shown in Figure 12, copying 100 copies and placing them in a certain folder, naming them according to (i), and using I values from 1 to 100 as the image number. We selected the batch image processing mode to calculate the classification and counting data of ferromagnetic particles. Table 3 lists a total of four rows of data from the test data in Figure 12.

The data results indicated that the repeated test data of the same sample were all the same, and there was no repeatability error in particle counting. A large number of samples were tested, and the results showed zero repeatability error in particle counting. This also reflected an advantage of image-based particle counting compared to shading based on particle counting.

According to empirical data partitioning, it can be divided into three intervals, such as 4–38 µm, 39–70 µm, and >70 µm.

### 4.2. The MTF Network

Wear usually occurs first with small particles, followed by large particles. The number of ferromagnetic particles is the most direct indicator for evaluating the wear condition of gears. The real-time monitoring characteristics of oil products mainly include three different sizes of ferromagnetic particles (4–38 µm, 39–70 µm, >70 µm). We consider the 4–38 µm- and the 39–70 µm-sized ferromagnetic particles as the health status, and the other monitoring characteristic data are selected as evaluation factors. The structure of the MTF network is outlined in Table 4.

#### 4.2.1. Wear Prediction Results of the MTF Network

**Figure 13:**
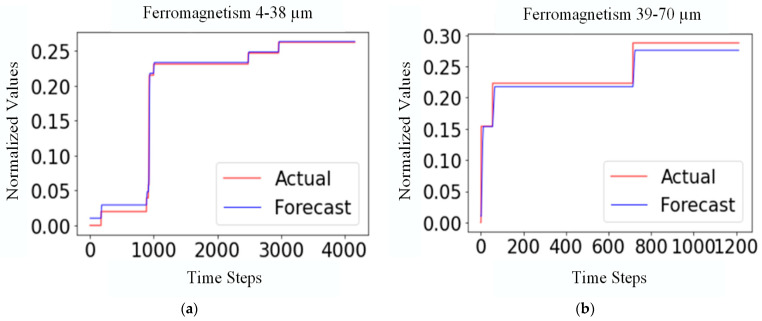
Prediction of the MTF model: (**a**) Ferromagnetism 4–38 μm and (**b**) Ferromagnetism 39–70 μm.

The proposed MTF network was used to make intelligent predictions of the data trend of Ferromagnetism 4–38 μm and Ferromagnetism 39–70 μm particles in oil. The input step size of the model was 50, the prediction step size was 1, the batch size was 250, and the epochs were 150. The logarithmic cumulative sum of the original data was preprocessed, and the model prediction results are shown in Figure 13.

Figure 13 shows that our proposed model can effectively predict the data trend of Ferromagnetism 4–38 μm and Ferromagnetism 39–70 μm particles in oil.

#### 4.2.2. Experimental Results and Analysis

To evaluate the performance of various intelligent prediction networks for time series data, we employ the classic LSTM and TCN methods for comparison and examine their respective predictive outcomes. The LSTM network is composed of a 1D convolution layer, LSTM layers, dense layers, and lambda layers, while the TCN network primarily consists of the input layer, TCN layer, and dense layer. The comparison results can be found in Table 5.

As illustrated in Table 5, the prediction errors for the MTF model are lower than those of the other two classic methods. The experimental outcomes demonstrate that the predictive performance of our proposed model surpasses that of LSTM and TCN, exhibiting outstanding generalization capabilities.

### 4.3. Data Consistency Analysis Verification

#### 4.3.1. Offline Sending of the Samples to the Laboratory for Comparative Verification and Analysis

On 29 November 2023, there was a sharp increase in particles of 4–38 µm and 39–70 µm. The system has an alarm reminder, with both interface alarm and sound alarm simultaneously. The data are shown in Table 6, Table 7 and Table 8.

In early December, there was a sharp increase in particles between 4–38 µm and 39–70 µm. The system has an alarm reminder, with both interface alarm and sound alarm simultaneously.

**Figure 14:**
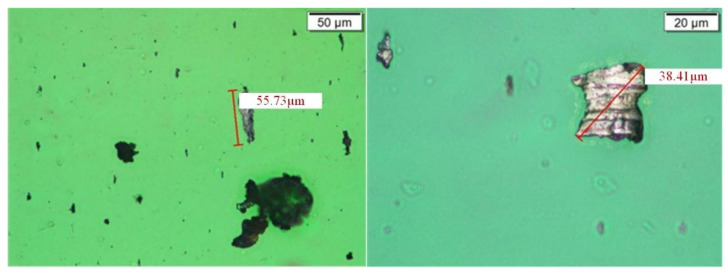
Wear particles in gearbox 15# (sampled on 3 December).

**Figure 15:**
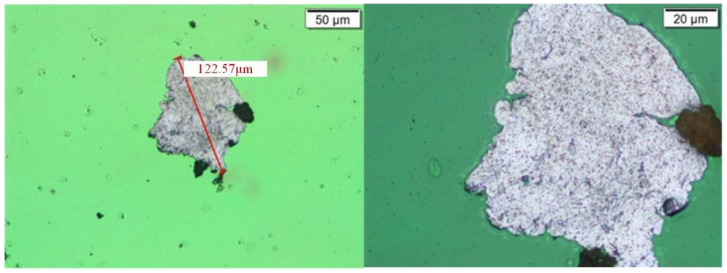
Wear particles in gearbox 15# (sampled on 12 December).

**Figure 16:**
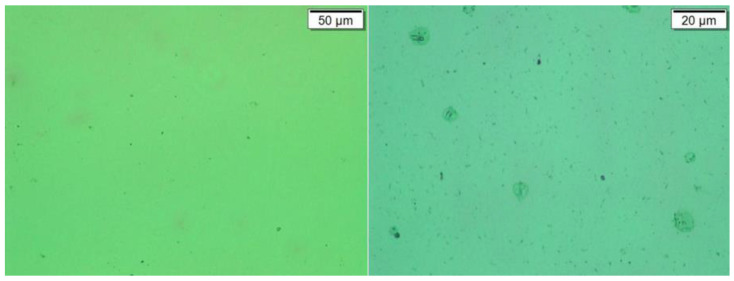
Wear particles in gearbox 15# (sampled on 14 December).

For this continuous alarm situation, the samples of gearbox lubricating oil were taken on 3 December, 12 December, and 14 December, and sent to the laboratory for offline inspection and analysis to verify the offline ferrography of the oil. The test results are shown in Figure 14, Figure 15 and Figure 16.

From the analysis of ferrography and high-power microscopy, it can be seen that there are indeed large-sized particles in the oil, with the maximum size reaching 120 μm. These large-sized particles are within the detection range of the sensor, so they can be captured by online monitoring devices, which also verifies the accuracy and reliability of online real-time monitoring.

#### 4.3.2. Disassembly and Maintenance

**Figure 17:**
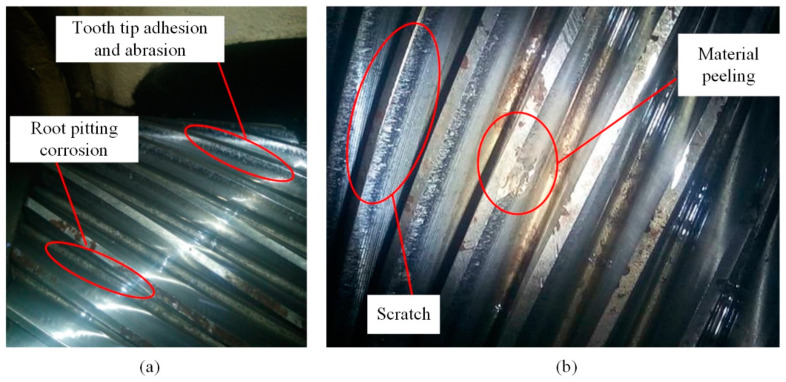
(**a**) Tooth tip abrasion and wear. (**b**) Wear of non-meshing surfaces.

Based on the particle morphology, disassembly, maintenance, and repair were carried out. Fatigue pitting on the tooth surface usually occurs near the tooth root, as this is usually a single tooth meshing area where the direction of friction changes and the tooth surface bears the maximum load. A certain part of the gear teeth captured on-site experienced severe fatigue below the pitch line of the tooth surface, but there was still a portion of the teeth that did not experience fatigue pitting in the root meshing area. However, severe abrasion and wear occurred at the tooth tips, and a large area of material peeling occurred at some tooth tips, as shown in Figure 17. This indicates that the tooth tips experienced severe adhesive wear and also suffered significant impact forces.

We determined that improper gear installation and adjustment was the root of the gear issue by conducting an analysis. The center distance of the gear transmission is too large or too small, or the bearings are installed incorrectly, resulting in biased load and incorrect meshing of the gears. This leads to fatigue pitting of some teeth below the pitch line adhesive abrasion and wear of some teeth at the tooth tip of high-speed gears.

## 5. Conclusions and Future Work

In this paper, we propose a method for diagnosing the wear condition of mechanical equipment based on online wear particle images. Firstly, a foreground segmentation preprocessing method based on the U-Net network can effectively eliminate the interference of bubbles and dark fields in online wear particle images. A total of 1960 wear particle images were collected in the experiment, and the average intersection union ratio of the validation set is 0.9299, and the accuracy of the validation set is 0.9799. Secondly, based on the foreground segmentation preprocessing of wear particle images, by using the watered algorithm to obtain the number of particles in each size segment, we obtained the number of magnetic particle grades in three different ranges: 4–38 µm, 39–70 µm, and >70 µm. Thirdly, we proposed a method named multidimensional transformer (MTF) network, MSE, RMSE, and MAE evaluation indexes are used to obtain the error, and the maintenance strategy is formulated according to the predicted trend. The experimental results show that the predictive performance of our proposed model is better than that of LSTM and TCN. Finally, the online real-time monitoring system triggered three alarms, and at the same time, our offline sampling data analysis was conducted three times, the accuracy of online real-time monitoring alarms was verified, and the equipment was disassembled for maintenance and repair.

In the future, we will describe the correlation between various features in online monitoring of oil through the spectrum and will fully combine the data of online monitoring and offline detection through multi-modal data fusion, to achieve accurate and early warning of the fault of the machine. The online wear state digital imaging excitation sensors designed for testing have the advantages of real-time and fast performance. However, under complex and ever-changing actual working conditions, most of them are easily affected by factors such as bubbles in the oil and equipment vibration, resulting in unclear images, compared to offline laboratory testing and analysis, most detection particle size ranges are still insufficient, and the collection of large-sized wear particles is not comprehensive when wear is severe, and the segmented wear particles are graded into different scales.

## Figures and Tables

**Table 1:** Equipment information table.

Equipment Installation Location	Guangdong Yuedian Power Plant				
Device name	15#, 24#, wind power gearbox	Lubrication oil	Mobil 320 gear oil	Lubricating system	Gearbox
Lubricating oil temperature	(60–65) °C	On-site temperature	−10 °C–45 °C	Pressure	0.1 Mba
Oil change interval	Offline inspection twice a year and oil change according to quality		Online detection indicators	Wear particle size distribution and particle images	

**Table 2:** Global average index.

Category	(Precision)	(Recall)	(IOU)
0 (background)	0.9890	0.9875	0.9768
1 (prospect)	0.9378	0.9448	0.8891

**Table 3:** Grading of wear particles.

Serial	Total Number of Wear Particles	4–6 μm	6–14 μm	14–21 μm	21–38 μm	38–70 μm	>70 μm	Coverage Area Ratio (%)
1	23	3	1	1	8	10	0	2.60391
2	23	3	1	1	8	10	0	2.60391
3	23	3	1	1	8	10	0	2.60391
…	…	…	…	…	…	…	…	…
100	23	3	1	1	8	10	0	2.60391

**Table 4:** Comprehensive structure of the MTF model.

MTF Model	
pos_encoder	PositionalEncoding ()
encoder	TransformerEncoderLayer
self_attn	MultiheadAttention
out_proj	LinearWithBias
Linear1/2	Linear
norm1/2	LayerNorm
dropout1/2	Dropout
transformer encoder	TransformerEncoder
ModuleList	TransformerEncoderLayer
self_attn	MultiheadAttention
out_proj	LinearWithBias
Linear1/2	Linear
norm1/2	LayerNorm
dropout1/2	Dropout
decoder	Linear

**Table 5:** The results of forecasting methods.

Method	MSE	RMSE	MAE
LSTM	0.004736	0.068817	0.066858
TCN	0.000156	0.012498	0.012073
MTF	3.458 × 10^−5^	0.005881	0.003568

**Table 6:** The measured data of 15# are as follows (corresponding to samples sent on 3 December).

Number	Time	Temperature	Ferromagnetism 4–38 µm	Ferromagnetism 39–70 µm	Ferromagnetism >70 µm	Flag
10	2023/11/29 6:55	78.39	19	10	1	1
9	2023/11/29 6:50	78.39	17	10	1	1
8	2023/11/29 6:45	78.29	17	7	1	1
7	2023/11/29 6:40	78.29	16	5	1	1
6	2023/11/29 6:35	78.39	13	5	1	1
5	2023/11/29 6:30	78.39	12	5	1	1
4	2023/11/29 6:25	78.39	12	5	1	1
3	2023/11/29 6:20	78.39	12	5	1	1
2	2023/11/29 6:15	78.39	12	5	1	1
1	2023/11/29 6:10	78.39	12	5	1	1
Average Value		78.39	14.2	6.2	1	1

**Table 7:** The measured data of 15# are as follows (corresponding to the sample sent on 12 December).

Number	Time	Temperature	Ferromagnetism 4–38 µm	Ferromagnetism 39–70 µm	Ferromagnetism >70 µm	Flag
10	2023/12/9 10:19	78.39	7	6	1	1
9	2023/12/9 10:14	78.39	6	5	1	1
8	2023/12/9 10:09	78.39	5	5	1	1
7	2023/12/9 10:04	78.39	5	4	1	1
6	2023/12/9 9:59	78.39	4	4	1	1
5	2023/12/9 9:54	78.39	4	4	1	1
4	2023/12/9 9:49	78.39	3	3	1	1
3	2023/12/9 9:44	78.39	3	2	1	1
2	2023/12/9 9:39	78.39	2	1	1	1
1	2023/12/9 9:34	78.39	2	1	1	1
Average Value		78.39	4.1	3.5	1	1

**Table 8:** The measured data of 15# are as follows (corresponding to the sample sent on 14 December).

Number	Time	Temperature	Ferromagnetism 4–38 µm	Ferromagnetism 39–70 µm	Ferromagnetism >70 µm	Flag
10	2023/12/13 10:19	78.39	5	1	1	1
9	2023/12/13 10:14	78.39	3	1	1	1
8	2023/12/13 10:09	78.39	2	1	1	1
7	2023/12/13 10:04	78.39	2	1	1	1
6	2023/12/13 9:59	78.39	2	1	1	1
5	2023/12/13 9:54	78.39	2	1	1	1
4	2023/12/13 9:49	78.39	2	1	1	1
3	2023/12/13 9:44	78.39	2	1	1	1
2	2023/12/13 9:39	78.39	2	1	1	1
1	2023/12/13 9:34	78.39	2	1	1	1
Average Value		78.39	2.4	1	1	1

## Data Availability

Data are contained within the article.
